# Evaluating Indicators of Continued Research Involvement and Activity in Hand Fellowship Faculty

**DOI:** 10.1016/j.jhsg.2026.101001

**Published:** 2026-03-27

**Authors:** Roshan V. Patel, Gnaneswar Chundi, Carl M. Harper, Tamara D. Rozental, Monica M. Shoji

**Affiliations:** ∗ Hand and Upper Extremity, Beth Israel Deaconess Medical Center, Harvard Medical School, Boston, MA; † Department of Orthopaedics, Rutgers New Jersey Medical School, Newark, NJ

**Keywords:** Fellowship, Hand, Productivity, Publications, Research

## Abstract

**Purpose:**

This study aims to evaluate indicators of continued research involvement and activity among hand fellowship faculty. Specifically, we assess the impact of research publication counts during different stages of medical training and geographic factors on the academic productivity of hand surgeons.

**Methods:**

A retrospective cross-sectional analysis was conducted using data from all fellowships listed on the American Society for Surgery of the Hand Fellowship Directory from June 2024 to July 2024. Faculty names were collected, and an algorithm was used to automate searches for research publication output across preresidency, residency, fellowship, and postfellowship periods. PubMed and Scopus databases were used to compile publication counts and H-indices. Data were categorized by geographic regions (Northeast, Midwest, South, West) and analyzed using descriptive statistics, Kruskal–Wallis tests, and negative binomial regression to determine the relationship between publication counts during training and total career publications.

**Results:**

The analysis included 94 hand fellowship programs and 645 physicians. Major regional differences were observed in publication counts during fellowship, postfellowship, and overall medical careers, with the Midwest showing the highest averages. A negative binomial regression revealed that publication counts during residency and fellowship, as well as the length of the medical career, independently predicted total career publications. Finally, Southern programs had the highest area deprivation index values, whereas Western programs had the highest Hirsch index to area deprivation index ratios, indicating increased productivity regardless of the area’s socioeconomic status.

**Conclusions:**

Research publication counts during hand surgeon training, particularly during fellowship, serve as key indicators of continued research leadership. Geographic variations suggest regional differences in research productivity and resource availability. These findings underscore the importance of early and sustained research involvement for academic success in hand surgery.

**Type of study/level of evidence:**

Prognostic III.

Research productivity markedly impacts residency and fellowship competitiveness and plays a key role in distinguishing future leaders and academic physicians across medical specialties. Orthopedic residents entering training with two or more publications publish considerably more during residency.^1^ Similarly, surgeons who completed formal research training later demonstrated higher publication counts and Hirsch index (H-index) values as faculty, illustrating the lasting impact of early research experiences.^2^ Concordant with physician performance, research involvement may serve as a proxy for dedication to the field of interest, leadership, and future academic engagement.

Among academic hand surgeons, a 2015 cross-sectional study found that the H-index, a widely accepted indicator of research impact, most strongly correlated with academic rank, and even served as a reliable predictor of current apointment.^3^ Furthermore, fellowship directors demonstrating an average H-index of 16.3.^4^ Through a rigorous publication record and sustained scholarly engagement, applicants can demonstrate commitment to the field and long-term academic potential.^5^

Although the orthopedic and spine literature indicate that research productivity during training predicts later academic output, subspecialty-specific data in hand surgery remain limited.^6^^,^^7^ Thus, the aim of this study is to characterize hand fellowship faculty productivity across training stages and regions, and evaluate whether publication counts during preresidency, residency, and fellowship predict career-long output.^8^ We hypothesize that higher research productivity throughout training correlates with increased research productivity across a hand surgeon’s career.

## Materials and Methods

A retrospective cross-sectional analysis was conducted across all hand surgery fellowships listed on the American Society for Surgery of the Hand Fellowship Directory webpage from June 2024 to July 2024.^9^ Hand fellowship faculty names were compiled for each listed program. Faculty were included regardless of primary residency training background, namely orthopedic surgery, plastic surgery, and general surgery. We then developed an automated algorithm to identify each faculty member’s research publication output before residency, during residency, during fellowship, and after fellowship. The algorithm scraped educational and professional data for surgeons from Doximity using each physician’s name, program, and state.

Once the educational data were collected, the algorithm searched PubMed for publications corresponding to each physician’s medical training stages. Using educational dates, the algorithm constructed search queries to find potential publications within specified ranges. The search results were then verified based on keywords extracted from the surgeon’s affiliations and professional experiences. Manual verification data were autonomously flagged and included publications that required human review because of partial matches or ambiguous affiliations. Publications that did not align with a surgeon’s verified affiliations or professional history were excluded. As this analysis had been conducted using publicly available data accessible through NCBI's PubMed, a publicly accessible database, we did not require Institutional Review Board approval.

Verified publication data were categorized as “preresidency verified,” “preresidency manual,” and “preresidency denied.” The total number of publications for each stage was also recorded. If a profile was not found on Doximity, the corresponding cells in the Excel summary were populated with “no profile.” Missing educational stages were labeled “no data” and flagged for manual verification.

Faculty H-indices were then retrieved from Elsevier’s Scopus database. Defined as the largest number of papers for which each article has been cited a certain number of times, the H-index may be calculated by listing an author’s publications in descending order by citations. Once completed, the number of papers whose position on the list is equal to or less than their number of citations is counted. The subsequent value is defined as an author’s H-index, a metric that is resistant to outlier publications that may have been cited many or a few times and is more representative of an author’s holistic career longitudinally.^10^

If complete educational histories could not be verified on Doximity, PubMed, LinkedIn, online resumes, or Scopus, they were excluded. Academic coordinators were not included. After compiling fellowship program data, each hand fellowship and affiliated faculty were assigned to a US region (Northeast, Midwest, South, or West) based on the Centers for Disease Control and Prevention (CDC) geographic region map.^11^ For publication counts, fixed inclusive ranges were established to account for research projects published between training periods. For a physician who graduated from medical school in 2008, for instance, all papers published in 2008 and before were considered preresidency publications, whereas all publications from 2009 to 2013 inclusive were considered residency publications. Publications released in 2014 were included in the fellowship range, and all publications in 2015 and beyond were classified as postfellowship publications. These training windows were applied consistently across all surgeons regardless of primary residency specialty. To calculate publication rates throughout training, research article counts were divided by the years spent in each training period, and postfellowship publication rates were similarly calculated using years since fellowship completion. Additionally, publication counts and rates throughout surgeons’ entire medical careers were collected, with a “medical career” defined as the number of years elapsed since beginning medical school.

Differences in residency length, as well as variations due to integrated versus independent residency structures, were not modeled separately. Dedicated research time, advanced degree training, or leave of absence during residency were not individually accounted for, as these intervals were not consistently identifiable across publicly available data sources.

As institutional funding data were not publicly available for many institutions listed, the area deprivation index (ADI) developed by the Health Resources and Services Administration (HRSA) was used. Quantified on a scale from 1 to 100, the ADI can best be defined as a composite value shaped by education, housing, poverty, employment, and US Census data, with higher ADI scores indicative of greater relative deprivation and lower ADI scores demonstrative of less disadvantaged areas.^12^ ADI was used to approximate the surrounding socioeconomic context of fellowship programs rather than as a direct measure of institutional funding or academic research infrastructure. For each program, the primary affiliated hospital Zone Improvement Plan (ZIP) code was queried in the University of Wisconsin School of Medicine and Public Health Neighborhood Atlas to obtain ZIP code-level national ADI percentile rankings, providing a geographically granular measure of regional socioeconomic context.^13^ If an ADI value was not available for a program, the ADI of the nearest region was selected. Consequently, H-index to ADI ratios were calculated, characterizing research output relative to surrounding socioeconomic status, in which higher scores indicate greater productivity despite greater area deprivation. These ratios were then compiled into a heat map, illustrating H-index to ADI ratios by state. Additional individual- and program-level characteristics, including residency accreditation pathway, fellowship size, surgeon board certification specialty, advanced degrees, and federal research funding, were not included because of inconsistent availability across publicly accessible data sources.

Descriptive and summary statistics were compiled by geographic region as well as nationally. Publication counts, rates, and surgeon experience statistics were compared using the nonparametric Kruskal–Wallis test. A negative binomial regression analysis was used to evaluate the association between publication counts during discrete training stages (preresidency, residency, and fellowship), years since starting medical school, and total career publication count, defined as the cumulative number of publications over a surgeon’s medical career. A significance value of *P* < .05 was selected for this study.

## Results

Ninety-four hand fellowship programs were included. A total of n = 645 physicians met inclusion criteria; n = 36 were excluded because of incomplete profile data. Of the 94 programs, 27 (28.7%) were located in the Northeast, 19 (20.2%) in the Midwest, 31 (33.0%) in the South, and 17 (18.1%) in the West (Table 1). Each attending surgeon included in this analysis had an average of 15.8 ± 11.5 years of experience following fellowship completion and maintained an H-index of 13.1 ± 13.7.

**Table 1:** Descriptive Data

Geographic Region	No. of Programs	No. of Physicians
Northeast	27	190
Midwest	19	142
South	31	203
West	17	110
US Total	94	645

Across all programs, the average surgeon published 33.1 ± 66.2 papers over the course of their medical career, with an average of 0.64 ± 2.83 publications during the preresidency period, 2.80 ± 6.79 publications during the residency period, 1.52 ± 3.74 publications during fellowship, and 28.1 ± 64.6 publications postfellowship. The number of publications during fellowship (*P* = .012), postfellowship (*P* = .004), and medical career (*P* = .002) significantly varied by geographic region, with the Midwest having the highest mean publication counts of 2.15 ± 4.05, 43.39 ± 107.40, and 49.94 ± 108.80 during each of the aforementioned training periods (Table 2).

**Table 2:** Mean (± SD) Publication Counts at Each Training Stage, Stratified by US Geographic Region

Time	US Total	Northeast	Midwest	South	West	*P* Value
Preresidency	0.64 ± 2.83	0.46 ± 2.10	0.91 ± 4.22	0.56 ± 2.57	0.72 ± 2.05	.115
Residency	2.80 ± 6.79	2.49 ± 7.61	3.49 ± 7.14	2.57 ± 5.92	2.88 ± 6.31	.207
Fellowship	1.52 ± 3.74	1.63 ± 4.65	2.15 ± 4.05	1.09 ± 2.76	1.33 ± 2.98	**.012**
Postfellowship	28.09 ± 64.63	32.56 ± 53.43	43.39 ± 107.40	17.25 ± 39.35	20.64 ± 36.39	**.004**
Medical career	33.06 ± 66.18	37.15 ± 55.19	49.94 ± 108.80	21.47 ± 41.41	25.56 ± 37.35	**.002**

Bolded values indicate statistical significance.

With respect to publication rate, hand surgeons nationally averaged 0.16 ± 0.71 papers per year in the preresidency period, 0.56 ± 1.36 in the residency period, 1.62 ± 4.48 in the fellowship period, and 1.84 ± 3.65 in the postfellowship period. Throughout the average hand surgeon’s medical career, an average of 1.33 ± 2.31 papers per year was found, as calculated by dividing their total number of publications by the number of years since beginning medical school. Mirroring observed trends in counts, publication rates significantly differed by US geographic region, during fellowship (*P* = .016), postfellowship (*P* = .005), and medical career periods (*P* = .002), with the Midwest maintaining the highest mean rates for each. In particular, the average Midwest hand surgeon maintained 2.15 ± 4.05 publications per year during their fellowship, 2.69 ± 5.02 publications per year postfellowship, and 1.90 ± 3.33 publications per year throughout their medical career (Table 3).

**Table 3:** Publication Rate by US Geographic Region

Time	US Total	Northeast	Midwest	South	West	*P* Value
Preresidency	0.16 ± 0.71	0.12 ± 0.53	0.23 ± 1.05	0.14 ± 0.64	0.18 ± 0.51	.115
Residency	0.56 ± 1.36	0.50 ± 1.52	0.70 ± 1.43	0.52 ± 1.18	0.58 ± 1.26	.217
Fellowship	1.62 ± 4.48	1.63 ± 4.65	2.15 ± 4.05	1.40 ± 5.21	1.33 ± 2.98	**.016**
Postfellowship	1.84 ± 3.65	2.00 ± 3.30	2.69 ± 5.02	1.30 ± 3.07	1.45 ± 2.81	**.005**
Medical career	1.33 ± 2.31	1.45 ± 2.19	1.90 ± 3.33	0.94 ± 1.73	1.08 ± 1.59	**.002**

Bolded values indicate statistical significance.

Besides publication count and rate, ADI (*P* < .001) as well as H-index/ADI ratios (*P* < .001) significantly varied across geographic regions, with Southern programs found to have the highest ADI values at 37.07 ± 21.55, and Western programs maintained the greatest H-index/ADI ratios of 2.32 ± 6.35. No significant differences were observed in surgeons’ years of postfellowship experience (*P* = .109) or H-index by geographic region (*P* = .202) (Table 4).

**Table 4:** Surgeon Experience and Research Productivity by US Geographic Region

Time	US Total	Northeast	Midwest	South	West	*P* Value
Postfellowship Years of Experience	15.83 ± 11.51	17.29 ± 11.92	14.07 ± 10.77	15.95 ± 11.54	15.39 ± 11.43	.109
H-index	13.09 ± 13.71	13.25 ± 11.38	15.33 ± 18.50	11.28 ± 11.92	13.29 ± 12.93	.202
ADI	32.43 ± 23.37	31.24 ± 29.60	34.60 ± 16.17	37.07 ± 21.55	23.12 ± 19.31	**<.001**
H-index/ADI	1.18 ± 3.09	1.64 ± 2.51	0.61 ± 0.92	0.53 ± 0.80	2.32 ± 6.35	**<.001**

Bolded values indicate statistical significance.

A negative binomial regression was conducted to analyze the association between publication counts during training stages and years since beginning medical school with total career publication count. More precisely, the publication counts during residency and fellowship and years since starting medical school were significantly associated with total career publications (residency incidence rate ratio [IRR] 1.034, *P* = 0.021; fellowship IRR 1.214, *P* < .001; career length IRR 1.078, *P* = .125) (Table 5).

**Table 5:** Association Between Training-Period Publication Counts and Total Career Publication Output∗

Variable	B	SE	IRR(Exo[B])†	Z	*P* Value
Preresidency Publication count	0.037	0.024	1.038	1.532	.125
Residency publication count	0.034	0.146	1.034	2.309	**.021**
Fellowship publication count	0.194	0.025	1.214	7.649	**<.001**
Medical career	0.075	0.006	1.078	12.621	**<.001**

Bolded values indicate statistical significance.

∗ Total career publication count represents the cumulative number of publications accrued over a surgeon’s medical career and includes publications during training and postfellowship practice.

† IRR indicates incidence rate ratio derived from the exponentiated regression coefficient (Exp[B]).

For every increase in the number of publications during residency by one, the total number of publications increased, on average, by 3.4% (*P* = .021). However, a larger effect of the number of publications during the fellowship period was observed; more specifically, for each increase in the number of publications during a surgeon’s fellowship, the total number of publications increased, on average, by 21.4% (*P* < .001). Lastly, for every increase in the number of years the surgeon has been active since medical school, the total number of publications increased on average by 7.8% (*P* < .001). Although there was a positive effect of the number of publications preresidency on the total number of publications, this impact was not statistically significant (*P* = .125) (Table 5).

Finally, a comprehensive heat map was generated to visualize the H-index/ADI ratios across state regions. The intensity of shading reflected various state-specific ratios, in which darker hues represented states exhibiting higher ratios and lighter hues indicated lower ones (Fig). The distribution of fellowship programs by state is provided in Table 6 for contextual interpretation of the heat map.

**Figure:**
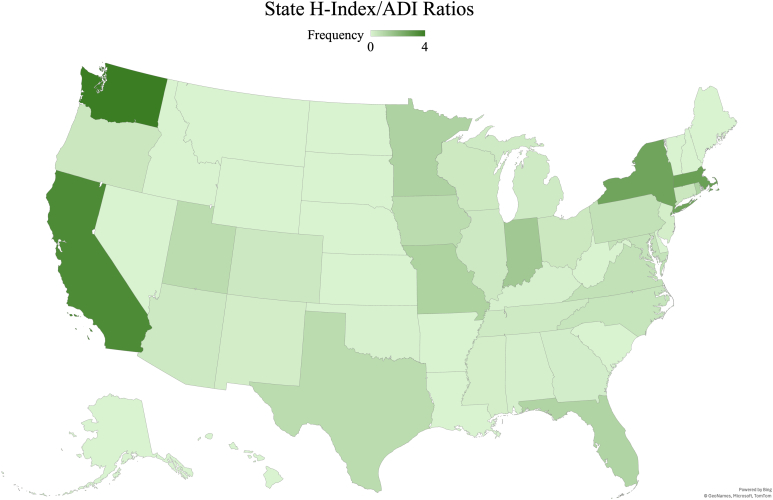
H-Index to ADI ratios by state.

**Table 6:** Fellowship Programs Per State

State	No. of Hand Fellowship Programs
Alabama	1
Arizona	1
California	11
Colorado	1
Connecticut	2
Florida	7
Georgia	1
Illinois	4
Indiana	2
Iowa	1
Kentucky	2
Maryland	4
Massachusetts	6
Michigan	2
Minnesota	2
Mississippi	2
Missouri	2
New Jersey	1
New Mexico	1
New York	9
North Carolina	3
Ohio	4
Oklahoma	1
Oregon	1
Pennsylvania	8
Rhode Island	1
Tennessee	3
Texas	3
Utah	1
Virginia	4
Washington	1
Wisconsin	2

## Discussion

Our results demonstrate that research productivity during fellowship had the strongest correlation with research productivity across a surgeon’s career, whereas preresidency output demonstrated no independent effect. Additionally, H-indices, publication count, and rate both notably differed by region during fellowship and postfellowship periods, as well as over the course of a surgeon’s career. Total career publication count was selected as the primary outcome to assess whether research productivity during training serves as a reliable indicator of sustained academic engagement across a surgeon’s career. Although postfellowship publication count represents an alternative outcome that avoids overlap with training-period predictors, the present approach aligns with prior bibliometric studies evaluating long-term academic productivity. Geographic region was examined as a contextual variable to explore broad patterns in academic productivity rather than as a substitute for individual surgeon-level or program-level characteristics. We do not suggest that geography is more informative than factors such as program structure, mentorship, or funding availability, which likely play a central role in shaping post-training productivity.

Our findings parallel those of Khawaja et al,^14^ who recently evaluated research productivity among 230 practicing hand surgeons and demonstrated that publication activity during medical school, residency, and fellowship was associated with later academic output and H-index. Consistent with their results, our analysis identified the fellowship period as the most powerful predictor of long-term productivity, followed by residency, whereas preresidency output demonstrated no independent effect after adjustment. The congruence between our studies reinforces that research engagement during the latter stages of training, namely fellowship, is a durable indicator of future output.^14^

Numerous distinctions extend the work of Khawaja et al. Our data set encompasses 94 accredited fellowships and 645 faculty members, representing a larger and exclusively academic cohort.^14^ Instead of dividing training intervals retrospectively, this analysis employed a priori, date-anchored publication windows verified through Doximity and PubMed, ensuring that training phases were not arbitrarily defined. Publication counts were modeled using negative binomial regression, which accommodates overdispersed count data. These methods revealed notable regional variation in both fellowship and postfellowship productivity, with the Midwest demonstrating the highest output. Moreover, by incorporating the ADI, this study introduces a socioeconomic dimension to academic productivity not previously explored in hand surgery literature.

Taken together, both investigations arrive at the conclusion that research involvement during fellowship is a marker of sustained academic involvement. Our larger analysis confirms this relationship within a broader national sample and contextualizes it across geographic and socioeconomic environments. These findings underscore the importance of mentorship and infrastructure within fellowship training.^14^

Our results demonstrated that research productivity during fellowship was the strongest predictor of long-term output. Productivity varied substantially by region, with hand surgeons in the Midwest maintaining the highest publication rates, H-indices, and output during fellowship and postfellowship periods.^15^ These findings mirror trends in other subspecialties, where surgeons trained in the Midwest similarly achieved the greatest research productivity during and after training, suggesting that regional factors, such as mentorship networks, balanced clinical and administrative responsibilities, and institutional cultures that prioritize scholarship, may foster sustained research engagement.^5^ These environments create a reinforcing cycle in which research-oriented surgeons recruit and mentor like-minded trainees. It should be noted, however, that this study was not designed to evaluate whether regional differences were driven by a small number of exceptionally high-output programs or reflected more uniform productivity across institutions within the Midwest. Future program-level or hierarchical analyses may help distinguish concentrated institutional effects from broader regional academic patterns.

This analysis is the first to examine wealth in relation to research productivity. ADI values varied greatly among hand fellowship programs, with Southern programs located in more socioeconomically deprived areas. Western programs demonstrated the highest H-indices relative to their ADI. This may reflect the strong link between medical school and residency training location, as programs in the US West South Central Division maintain the highest proportion of residents matching within the same census division.^16^ This geographic continuity likely extends to fellowship training, fostering local networks that enhance research output, even in regions with lower socioeconomic status or limited funding.

Lambrechts et al^7^ reported that high publication rates during training predicted greater future productivity among orthopedic and neurosurgical spine surgeons, whereas preresidency publications were poor predictors of attending output. However, Goss et al^6^ observed that publications during medical school, residency, and fellowship correlated with attending productivity during the first 5 years of practice, though this relationship diminished over time. Likewise, Rompala et al^5^ found that research during all training phases was associated with higher postfellowship output, with fellowship work showing the strongest predictive value and preresidency research retaining some influence.

These studies propose that although early research can predict short-term productivity, its long-term significance is less consistent. The mixed association reflects that preresidency output is shaped by access and opportunity over interest.^6^ As residency selection becomes more competitive, applicants may pursue research primarily to strengthen their applications, weakening its predictive value.^17^ Thus, early productivity should be interpreted cautiously despite its current weight in admissions. Longitudinal assessment of evolving research attitudes may provide a more meaningful measure of academic potential.^18^

One distinguishing feature of hand fellowships is their diverse applicant pool. Unlike other orthopedic subspecialties, many hand fellowships accept plastic and general surgery applicants. In 2018, 68% of fellows came from orthopedic residencies, 28% from plastics, and 4% from general surgery. Plastic surgery residents demonstrated higher citation counts, publication totals, and H-indices than orthopedic residents.^15^

There are several limitations to our study. First, although ADI was assigned at the hospital ZIP code level, substantial heterogeneity in deprivation can exist within states and metropolitan areas. Neighborhood-level ADI may not capture institutional-level resources such as protected research time, grant funding, or administrative support, all of which can substantially influence academic productivity. Therefore, ADI should be interpreted as a contextual socioeconomic marker rather than a direct proxy for academic medical center research capacity.

Additionally, publication counts may be underestimated for surgeons who changed surnames during their careers, particularly women who changed names because of marriage or other personal reasons. Because maiden names and previous surnames could not be systematically identified using public data, publications authored under alternate names may not have been captured, introducing potential misclassification bias.

There may also be additional factors that impact research productivity that we were unable to take into account. For example, in the primary care setting having access to an “influential mentor” resulted in 5.0 greater odds of publishing more than one article per year.^19^ These findings highlight the significance of program-specific dynamics as well as the impact of personal and research relationships made during medical training on research publication output. Secondly, we were unable to account for dedicated research periods during training. However, we believe that this would not meaningfully affect our results as a 2017 comparative study of three orthopedic residency programs found that programs involving a designated research period failed to produce an appreciably larger number or higher caliber research publications than orthopedic residency programs without such a period.^20^ The referenced orthopedic residency programs serve as illustrative examples of research productivity patterns; however, the duration of dedicated research training within these programs (short duration versus more than one year) was not differentiated in our analysis.

Similarly, training timelines did not account for specialty-specific residency length, advanced degree training, or other leave of absence periods during the residency period itself. These factors may have resulted in potential misclassification of publications across training stages, particularly among plastic surgery-trained surgeons, but are unlikely to materially alter the observed relationship between fellowship-period productivity and long-term academic output.

Moreover, although the H-index may serve as a reliable measure, it is not immune to manipulation by researchers and authors. As such, excessive self-citation may be used to inflate one’s research prominence and is not reflected in H-index values.^21^ However, this should have minimal impact on the applicability of our findings.

Finally, because the total career publication count includes publications completed during training, the modeled outcome necessarily overlaps with training-period predictors. Although this approach was chosen to evaluate long-term academic productivity, future analyses may benefit from solely modeling postfellowship publications to isolate attending-level output.

In summary, research productivity particularly in fellowship was markedly predictive of as increased publication rate as an attending hand surgeon. Programs seeking to cultivate academic surgeons should interpret preresidency publications cautiously while prioritizing structured mentorship during residency and fellowship training periods.

## Conflicts of Interest

Dr Rozental is a consultant for Stryker and Teladoc Health, and is the past President of the American Society for Surgery of the Hand. No benefits in any form have been received or will be received by the other authros related directly to this article.
